# Isolation of *Leptospira interrogans* serovar Hardjoprajitno from a calf with clinical leptospirosis in Chile

**DOI:** 10.1186/s12917-015-0369-x

**Published:** 2015-03-18

**Authors:** Miguel Salgado, Barbara Otto, Manuel Moroni, Errol Sandoval, German Reinhardt, Sofia Boqvist, Carolina Encina, Claudia Muñoz-Zanzi

**Affiliations:** Department of Biochemistry and Microbiology, Faculty of Sciences, Universidad Austral de Chile, Edificio Instapanel, Campus Isla Teja, CC 567 Valdivia, Chile; Department of Animal Pathology, Faculty of Veterinary Sciences, Universidad Austral de Chile, Valdivia, Chile; Department of Biomedical Sciences and Veterinary Public Health, Swedish University of Agricultural Sciences, Box 7028, SE-750 07 Uppsala, Sweden; Division of Epidemiology and Community Health, School of Public Health, University of Minnesota, Minneapolis, Minnesota USA

**Keywords:** *Leptospira*, Cattle, Clinical, Hardjoprajitno

## Abstract

**Background:**

Although *Leptospira* isolation has been reported in Chilean cattle, only serological evidence of serovar Hardjo bovis infection has been routinely reported. The present report provides characterization of the pathological presentation and etiology of a clinical case of leptospirosis in a calf from the Los Rios Region in Chile.

**Case presentation:**

In a dairy herd in southern Chile, 11 of 130 calves died after presenting signs such as depression and red-tinged urine. One of these calves, a female of eight months, was necropsied, and all the pathological findings were consistent with *Leptospira* infection. A urine sample was submitted to conventional bacteriological analysis together with highly specific molecular biology typing tools, in order to unravel the specific *Leptospira* specie and serovar associated with this clinical case.

A significant finding of this study was that the obtained isolate was confirmed by PCR as *L. interrogans,* its VNTR profile properly matching with *L. interrogans* Hardjoprajitno as well as its specific genomic identity revealed by *secY* gen.

**Conclusion:**

*Leptospira interrogans* serovar Hardjoprajitno was associated with the investigated calf clinical case.

This information adds to the value of serologic results commonly reported, which encourage vaccination improvements to match circulating strains. In addition, this finding represents the first case report of this serovar in Chilean cattle.

## Background

Leptospirosis is a zoonotic infectious disease caused by bacteria of the genus *Leptospira* that affects domestic and wild animals. The disease is distributed worldwide and of great public health importance, especially in warm and humid climates. The bacterium is shed in the urine of infected animals and this is the main transmission route for human infection [1]. The disease has also been recognized as one of the most important diseases in livestock, particularly in cattle, due to negative impacts on reproduction [2,3].

It is well established that *Leptospira* infection in Chile is present both in domestic and wild animals [4,5]. The apparent seroprevalences in different domestic animal species are high, ranging from 59 to 91% in cattle, 24% in goats, 7.1% sheep, 49% in equine, 70% in swine and 47% in wild mice [5]. In a study from Southern Chile 162/361 (45%) serum samples from apparently healthy cattle were seropositive for *Leptospira* using the Microscopic Agglutination Test. The proportion of seropositive samples was highest for *Leptospira* serovar Hardjo (68%), followed by serovars Pomona (11%), Tarassovi (8.6%), Bratislava (1.9%), Canicola (1.9%), Icterohaemorrhagiae (1.9%) and Ballum (1.2%) [4]. Recently, a study was carried out to determine *Leptospira* seroprevalence and to evaluate risk factors associated with seropositivity at herd level in smallholder bovine dairy herds in southern Chile, and 75% of the included herds (52/69) showed serological titers against one or more *Leptospira* serovar, where *Leptospira borgpetersenii* serovar Hardjo was the serovar most frequently (81%) reported from animals with positive results [6].

Infection by *L. interrogans* serovar Hardjo (type Hardjoprajitno) in cattle has not been investigated previously in Chile. The importance of serovar Hardjoprajitno infection on the rate of abortion has been estimated to be 30% as opposed to what happens with *Leptospira borgpetersenii* Hardjo bovis where the rate reaches only a 3 to 10%. Additionally, acute infection of dairy cows with Leptospira *interrogans* Hardjoprajitno is associated with a drop in milk production [7]. Therefore, the aim of the present study was to present both pathological and microbiological evidence of the *Leptospira interrogans* Hardjoprajitno virulence from its isolation and characterization from a calf that died of clinical leptospirosis.

## Case presentation

### Study animal

In a dairy herd in southern Chile, eleven out of 130 calves died after presenting clinical signs such as depression and haematuria. One of these calves, a female of eight months, was submitted to the Department of Animal Pathology at the Faculty of Veterinary Science, Universidad Austral de Chile, Valdivia, Chile for necropsy.

### Pathological findings

**Figure 1:**
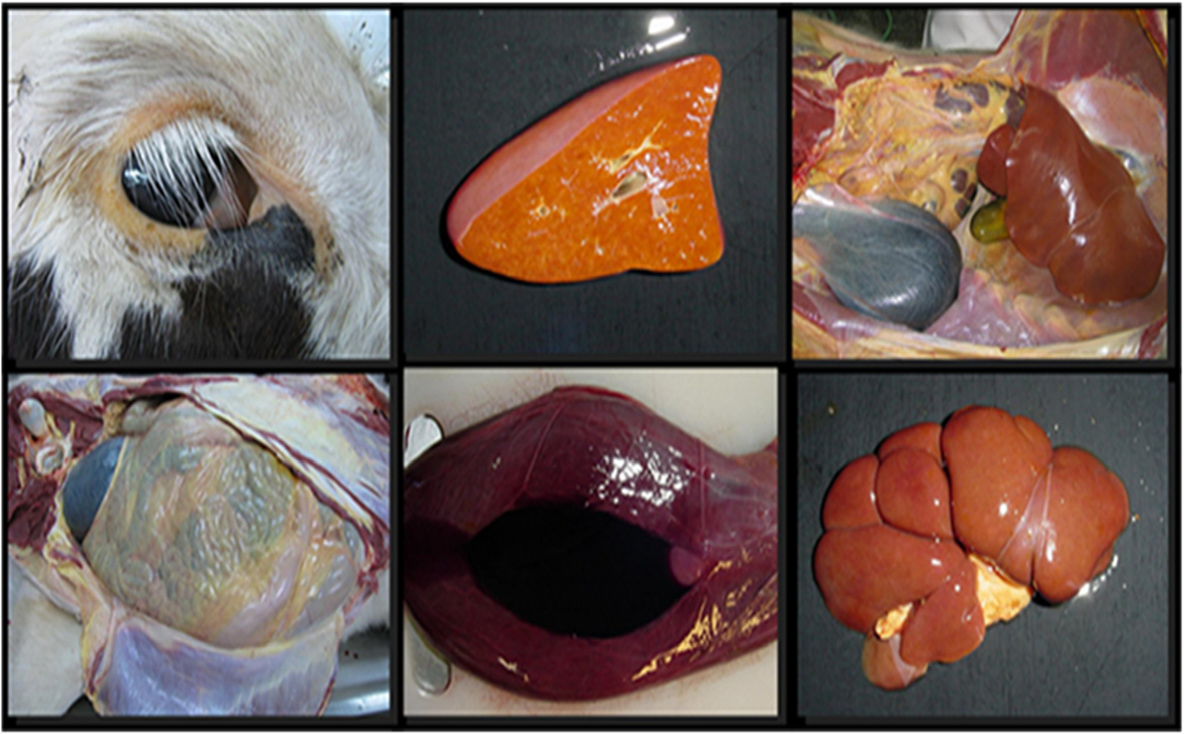
**Calf with clinical sign and pathological findings consistent with** ***Leptospira*** **infection.**

Necropsy showed a marked yellowing pigmentation in all mucosal body openings and in the subcutaneous tissue, fat and muscles. There were also isolated petechia in the kidneys and the bladder contained approximately two liters of red-tinged urine (Figure 1).

A urine sample was collected by puncturing the bladder and sent to the Leptospirosis and Paratuberculosis Laboratory, Department of Biochemistry and Microbiology, at the Faculty of Sciences, Universidad Austral de Chile, Valdivia, Chile. All the procedures were in strict accordance with the recommendations in the Guide of Use of Animals for Research of Universidad Austral de Chile, approved by the Committee on the Ethics of Animals for Research (www.uach.cl/direccion/ investigacion/uso_animales.htm).

### Bacteriological analysis

The urine sample was investigated using dark field microscopy and bacterial structures consistent with *Leptospira* were found. Thereafter, 200 μl of urine sample with four replicates was cultured in EMJH medium at 29°C [8]. After a month of incubation, a positive result was reported with a typical Dinger ring growth.

*Leptospira* DNA was extracted from positive cultures and in order to identify *Leptospira* species, primers covering the most common pathogenic *Leptospira* species were used (664–665 *L. kirschneri* fla gene; 1280–1281 *L. interrogans* IS1500; 1805–1809 *L. borgpetersenii* IS1533) [9]. The total PCR reaction was 50 μl, of which 5 μl was 10× Taq polymerase buffer (Promega, Madison, WI), 2 μl dNTPs (2.5 mM stock containing all four dNTPs) (Promega, Madison, WI), 0.5 U Taq Polymerase (Promega, Madison, WI), 1 μl (each) primer (stock concentration = 100 pmol/μl; final concentration 2 pmol/μl), 35.5 μl dH_2_O and 5 μl template. The PCR reactions considered 40 cycles of 94°C for 15 sec; 60°C for 30 sec and 68°C for 2 min. then 10°C hold. Negative and positive PCR controls were included as well as DNA extraction negative and positive controls.

To refine our understanding of the *Leptospira* specie and serovar associated with this clinical case, a Variable Number Tandem Repeat (VNTR) analysis was done. The VNTR primers were designed exclusively for use with *L. interrogans* [10-12]. PCR products for VNTR loci 4, 7, 10, 23, 27, 29, 30, 31, and 36 were assessed using the same PCR reaction as described above and the primers used were as previously reported [12]. PCR products were separated by agarose gel electrophoresis and visualized, and their sizes were calculated by comparing with reference standards (100-bp ladder; Invitrogen, Carlsbad, CA) and with the literature [10,11]. As a complement, we also amplified the gene *secY*, which is a house keeping gene that consists of alternating conserved and variable regions, making it suitable to deduce primers that generate amplicons with sufficient sequence heterogeneity to enable phylogenetic interpretation for *Leptospira* [13]. A 202 bp product was amplified by conventional PCR in 25 μl mixture containing 5 μl diluted template (1:100), 0.2 μM each primers SecYIVF (5′-GCGATTCAGTTTAATCCTGC-3′) and SecYIV (5′-GAGTTAGAGCTCAAATCTA-AG-3′), 0.625 U GoTaq Flexi DNA Polymerase in 1X Green Buffer GoTaq (Promega, Madison, WI), 3.0 mM MgCl2, 0.3 mM dNTPs (Promega, Madison, WI), and 400 ng mL-1 bovine serum albumin (BSA; BioLabs, Ipswich, England). Cycle conditions included an initial denaturation step at 95°C for 5 min followed by 40 cycles at 94°C for 1 min, 57°C for 1 min and 72°C for 1 minute and a final elongation step at 72°C for 10 minutes. The PCR products obtained were separated on 1.5% agarose gel, stained with Gel Red (GelRed, Biotium Inc, Hayward, U.S), excised and purified using a commercial kit (E.Z.N.A® Gel Extraction Kit, Omega Bio-Tek, Norcross, U.S). Amplicons were sequenced by Macrogen Inc (Seoul, Korea). The consensus nucleotide sequence obtained in this study was compared with *secY* gene of *Leptospira interrogans* serovar Hardjo prajitno (GenBank accession number EU357983.1). DNA alignments were done using clustalW tools (http://www.ebi.ac.uk/Tools/msa/clustalw2).

## Results and discussion

**Figure 2:**
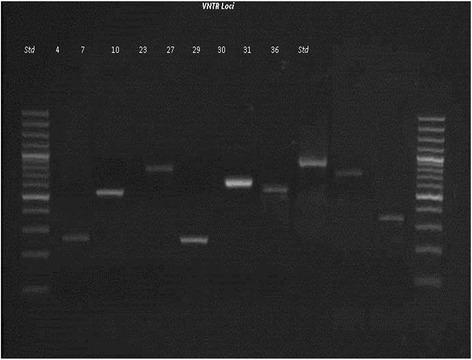
**PCR analysis of the polymorphism of nine representative VNTR loci.** Amplification was performed on the VNTR 4, 7, 10, 23, 27, 29, 30, 31, 36 loci of *L. interrogans* strains.

**Figure 3:**
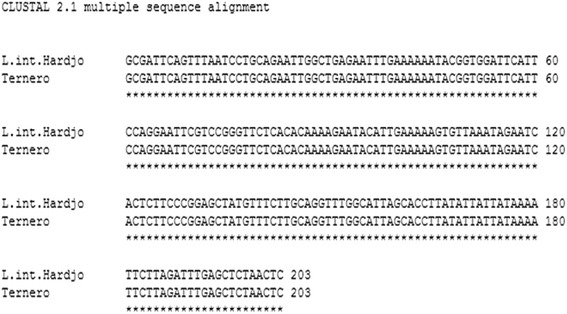
**ClustalW aligment for 202 bp fragment,** ***SecY*** **gen.**

The isolate obtained in this study was confirmed by PCR as *L. interrogans* and its VNTR profile properly matched with *L. interrogans* type Hardjo prajitno (Figure 2). The *secY* gene alignment done by clustalW did reveal sequence identity strain belonging to the species *L. interrogans* serovar Hardjo prajitno (Figure 3). This confirms that *L. interrogans* type Hardjo prajitno is associated with acute infection of cattle in Chile. Previous studies have shown that the abortion rate after *Leptospira borgpetersenii* serovar Hardjo bovis infection is 3 to 10% whereas the rate increases up to 30% for *L.* Hardjo prajitno infection [3-7], which underscores the importance of this serovar. The clinical information in the presented study was conveniently complemented with bacteriological findings to describe the isolated strain affecting the clinical case presented.

The typing method used based on VNTR polymorphism provided a rapid characterization together with the highly discriminatory power reported [10,11] to identify *L. interrogans* serovars using clinical specimens. Furthermore, the use of *secY* gene in combination with the latter allowed a more robust result due to its great phylogenetic potential [13].

## Conclusions

The present finding represents the first isolation confirmed as *L. interrogans* serovar Hardjo prajitno from cattle with clinical disease in Chile. The importance of this serovar in Chilean cattle needs to be investigated further. This information add to the value of serologic results commonly reported, which encourage vaccination improvements to match circulating strains. The latter is based on a previous published study on serological cross-reactivity between Hardjo bovis and Hardjoprajitno serovars, which implies similar antigenic determinants; although with substantial genomic differences as well as in their pathogenicity in the bovine specie [14].

Due to above mentioned, we emphasize the need to isolate, preserve and characterized strains of *Leptospira* in order to improve and standardize currently available diagnostic techniques. This will help to improve our understanding of the epidemiology and impact of this infection as well as to identify optimal option for surveillance and control.
